# Transradial versus transfemoral access in uterine artery embolization for fibroids: a systematic review and meta-analysis

**DOI:** 10.3389/fmed.2026.1718480

**Published:** 2026-03-06

**Authors:** Zainah Abdulbari Alhebshi, Marwah Nasir Ahmad, Mariam Amro Alsayed, Layan Hassan Aljarari, Maya Rawah, Mahnoor Hayat, Jina Khalid Mohammed Fadl, Ibrahim Daoud, Omnia Orabi Mohammed Orabi, Salma Mohammed Hassan Eltayeb

**Affiliations:** 1 General Medicine Practice Program, Batterjee Medical College, Jeddah, Saudi Arabia; 2 Department of Obstetrics and Gynecology, General Medicine Practice Program, Batterjee Medical College, Jeddah, Saudi Arabia; 3 Faculty of Medicine, University of Khartoum, Khartoum, Sudan; 4 Department of Obstetrics and Gynecology, General Medicine Practice Program, Batterjee Medical College, Aseer, Saudi Arabia; 5 Faculty of Medicine, Al Neelain University, Khartoum, Sudan; 6 Faculty of Medicine, Suez Canal University, Ismailia, Egypt; 7 Department of Obstetrics and Gynecology, Tawam Hospital, AlAin, United Arab Emirates

**Keywords:** interventional radiology, minimally invasive therapy, transfemoral access, transradial access, uterine artery embolization, uterine fibroids

## Abstract

**Background:**

Uterine artery embolization (UAE) is a well-established, minimally invasive treatment for symptomatic uterine fibroids. Traditionally performed through transfemoral access (TFA), there has been growing interest in the transradial approach (TRA) due to its reported benefits in other interventional procedures. This systematic review and meta-analysis aim to compare TRA and TFA in UAE for uterine fibroids.

**Methods:**

A systematic search of PubMed, Ovid MEDLINE, and Google Scholar was conducted from their date of inception to April 2025. Data were synthesized and analyzed using Review Manager. Risk of bias was assessed using the MINORS and RoB 2 tools.

**Results:**

Six studies (*n* = 639 patients) met the inclusion criteria. Of these, 324 (50.7%) patients were assigned to the TRA group, with a mean age of 43.48 ± 6.23 years, and 315 (49.3%) patients to the TFA group, with a mean age of 42.97 ± 6.33 years. TRA was associated with significantly lower radiation exposure (MD = −207.54 mGy, 95% CI [−262.42, −152.67], *p* < 0.00001), shorter procedure time (MD = −7.38 min, 95% CI [−10.09, −4.66], *p* < 0.00001), and a greater likelihood of same-day discharge (RR = 9.50, 95% CI [3.76, 24.03], *p* < 0.00001). TRA also showed fewer access-site complications (RR = 0.55, 95% CI [0.31, 0.97], *p* = 0.04), particularly hematomas (RR = 0.32, 95% CI [0.14, 0.74], *p* = 0.007). No significant difference was found in fluoroscopy time. Moreover, the TRA failure rate was low (0.3%).

**Conclusion:**

TRA is a safe and effective alternative to TFA for UAE in selected settings, providing reduced radiation exposure, shorter procedure times, faster recovery, and fewer access-site complications.

**Systematic review registration:**

https://www.crd.york.ac.uk/PROSPERO/view/CRD420251027231, CRD420251027231.

## Introduction

Uterine fibroids, also known as leiomyomas, are the most prevalent benign gynecological tumors in women of reproductive age. These smooth muscle neoplasms can vary in size and location, and while often asymptomatic, they can cause significant morbidity (1). Symptomatic fibroids are associated with heavy or prolonged menstrual bleeding, pelvic pain or pressure, urinary frequency, and infertility (2). These symptoms can significantly impair quality of life and frequently prompt women to seek medical or surgical intervention (3, 4).

For those wishing to preserve fertility or avoid major surgery, minimally invasive, uterus-sparing treatments have gained popularity. Among these treatments, uterine artery embolization (UAE) has been recognized since the early 1990s as an effective alternative to hysterectomy for symptom relief (4, 5). UAE entails catheter-directed embolization of the uterine arteries to induce ischemic shrinkage of fibroids (6) and has demonstrated favorable outcomes in terms of efficacy, recovery time, and uterine preservation (5).

Traditionally, UAE has been performed through the transfemoral approach (TFA), which provides access to the uterine arteries through the femoral artery (4). However, increasing attention has been directed toward the transradial approach (TRA), a technique originally developed for coronary interventions, which utilizes the radial artery at the wrist. The TRA offers several advantages over the TFA, including shorter recovery time, earlier ambulation, greater patient comfort, and reduced vascular complications (5, 7). These benefits have led to its growing use in non-cardiac interventions such as UAE.

Despite these advancements, comparative data between the TRA and the TFA in the context of UAE remain limited. Existing studies have assessed radiation exposure, complication rates, procedural success, and patient satisfaction, but they often have small sample sizes and inconsistent findings (7).

This systematic review and meta-analysis aimed to synthesize the available evidence comparing the TRA and the TFA in UAE, focusing on procedural efficacy, safety outcomes, and patient-centered outcomes. The findings may help inform clinical decision-making and enhance individualized patient care.

## Materials and methods

### Literature review

Our review was conducted using Cochrane review methods and followed the Preferred Reporting Items for Systematic Reviews and Meta-Analyses (PRISMA) guidelines (8, 9). This research adhered to the International Prospective Register of Systematic Reviews (PROSPERO) statement (ID: CRD420251027231) (10).

In April 2025, a comprehensive literature search was conducted in the PubMed, Ovid MEDLINE, and the Cochrane Library databases to identify studies comparing the TRA and the TFA for UAE. The search strategy combined controlled vocabulary and free-text terms related to the population and intervention, including “uterine artery embolization,” “uterine fibroid embolization,” “transradial,” “radial artery,” “transfemoral,” and “femoral artery.” No restrictions were applied to the publication date.

Reference lists of the included studies and relevant reviews were manually screened to identify additional eligible articles. Two reviewers independently screened titles and abstracts, followed by full-text assessments of potentially eligible studies. Disagreements were resolved by consensus to minimize selection bias and ensure comprehensive identification of all relevant evidence.

### Study selection

Studies were included if they were published at any time up to April 2025, reported the number of patients assessed, were published in English, compared the TRA and the TFA in the context of UAE for the treatment of uterine fibroids, and reported at least one clinically relevant outcome of interest. Eligible study designs included randomized controlled trials and comparative observational studies (prospective or retrospective).

Studies were excluded if they were published in languages other than English, did not compare the TRA and the TFA in the context of UAE for uterine fibroids, failed to report outcomes relevant to the clinical question, or were non-original designs, including case reports, systematic reviews, meta-analyses, narrative reviews, or scoping reviews.

This systematic review was designed according to the Population, Intervention, Comparison, Outcomes, and Study design (PICOS) framework. The population comprised women undergoing UAE for uterine fibroids. The intervention of interest was the TRA, and the comparator was the TFA. The outcomes of interest included radiation exposure parameters (peak skin dose and fluoroscopy time), procedural metrics (procedure duration and contrast volume), access-site complications, and discharge outcomes.

### Screening and data extraction

Full-text articles of studies that met the inclusion criteria were retrieved and screened for eligibility. Two authors independently evaluated the studies. In cases of disagreement, the lead author was consulted to resolve the issue and finalize the inclusion or exclusion decision. The entire screening process was documented using the PRISMA flow diagram.

Data extraction was performed independently by two authors using a standardized spreadsheet. Extracted variables included patient age and sex distribution, total sample size, fluoroscopy duration, radiation exposure metrics, access-site complications, procedural details, including crossover from the TRA to the TFA, and same-day discharge rates. In cases of disagreement, the lead author adjudicated. When essential data were missing, the corresponding authors were contacted; studies were excluded if no response was received.

### Bias assessment

Two reviewers independently assessed the risk of bias using the Methodological Index for Non-Randomized Studies (MINORS) for observational studies and the Revised Cochrane Risk of Bias Tool (RoB 2) for randomized controlled trials. A third reviewer evaluated all assessments to ensure consistency. The MINORS tool was used according to the study design: 8 domains were assessed for non-comparative studies (maximum score of 16), and 12 domains were assessed for comparative studies (maximum score of 24), with each domain scored from 0 to 2 (11). For randomized controlled trials, the RoB 2 tool was used, which evaluates five domains related to the trial’s design, conduct, and reporting. Each domain includes structured signaling questions that guide the overall judgment. Based on the responses, studies were categorized as having a low risk of bias (all domains at low risk), some concerns (one or more domains with some concerns), or a high risk of bias (one or more domains at high risk) (12).

### Statistical analysis

The meta-analysis was performed using Review Manager (RevMan) software, developed by the Cochrane Collaboration. Data were extracted from the selected studies by four authors, and the statistical analysis was supervised by an author with expertise in statistics. The analysis involved calculating standard mean differences (MDs) or risk ratios (RRs), reported with 95% confidence intervals (CIs). To assess variability between the studies, the chi-squared and I-squared tests were performed using RevMan. If significant heterogeneity was detected (*I*^2^ > 50%), a random-effects model was used; otherwise, a fixed-effects model was used. Any disagreements that emerged during data interpretation were resolved through team discussion to reach consensus. When necessary, the original study authors were contacted to obtain missing information.

## Results

### An overview of the reviewed studies’ characteristics

**Figure 1:**
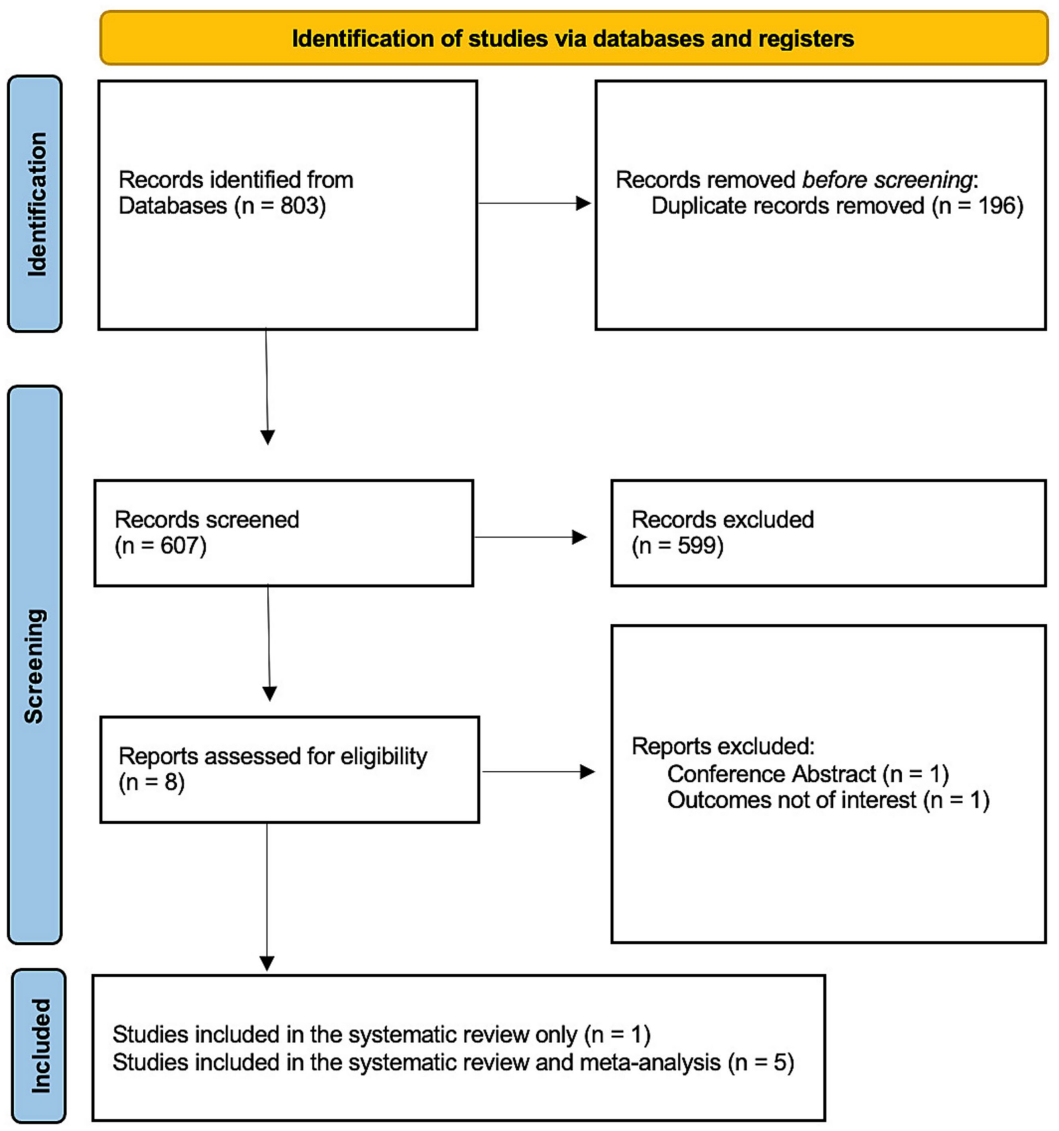
Flowchart outlining the selection of the reviewed studies according to the preferred reporting items for systematic reviews and meta-analysis (PRISMA) guidelines.

A comprehensive search of primary databases, such as PubMed, Ovid MEDLINE, and Google Scholar, initially yielded 803 records. Following rigorous screening and an eligibility assessment, eight studies were shortlisted, six of which met the predefined inclusion criteria (Figure 1). These included two randomized controlled trials (RCTs), three retrospective studies, and one prospective study (13–18). Collectively, the six studies included a total of 639 female patients: 324 (50.7%) were assigned to the TRA group, with a mean age of 43.48 ± 6.23 years, and 315 (49.3%) were assigned to the TFA group, with a mean age of 42.97 ± 6.33 years (Table 1). Notably, seven patients in the TRA group and two in the TFA group presented with fibroids associated with adenomyosis (18). Additionally, four patients in the TRA group and three in the TFA group had postpartum hemorrhage and were managed using UAE (16).

**Table 1:** Characteristics of the included studies.

Article	Country	Study design	Total participants, *N*	Participants grouping		Age (years), mean ± SD (range)		BMI (Kg/m^2^), mean ± SD		Height (cm), mean ± SD		Associated adenomyosis	
				TRA	TFA	TRA	TFA	TRA	TFA	TRA	TFA	TRA	TFA
Bessar et al. 2025 (13)	Egypt	RCT	42	21	21	44.86 ± 5.14	43.86 ± 4.10	29.77 ± 3.51	28.30 ± 3.85	166.19 ± 9.14	163.10 ± 7.01	NR	NR
Ghosh et al. 2022 (14)	United States	Retrospective study	172	96	76	43.2 ± 7.2	43.7 ± 7.2	NR	NR	NR	NR	NR	NR
Gjoreski et al. 2019 (15)	North Macedonia	Retrospective study	24	11	13	38.5 ± 5.6	38.5 ± 4.1	NR	NR	NR	NR	NR	NR
Khayrutdinov et al. 2020 (16)	Russia	RCT	153	78^a^	75^b^	40.42 ± 5.32	39.08 ± 5.88	26.15 ± 3.57	25.83 ± 3.89	169.16 ± 9.23	167.21 ± 8.72	NR	NR
Mortensen et al. 2018 (17)	United Kingdom	Prospective study	66	27	39	45.1 ± 4.9	44.4 ± 4.9	NR	NR	NR	NR	NR	NR
Nakhaei et al. 2019 (18)	United States	Retrospective study	182	91	91	46.2 ± 4.9	45.4 ± 5.4	31.5 ± 9.8	29.7 ± 7.6	164 ± 6.8	165.0 ± 6.9	7	2

a Four patients had postpartum hemorrhage only.

b Three patients had postpartum hemorrhage only.

TRA, transradial artery; TFA, transfemoral artery; RCT, randomized controlled trial; USA, United States of America; UK, United Kingdom; BMI, body mass index; NR, not reported.

### Radiation exposure (mGy)

**Figure 2:**
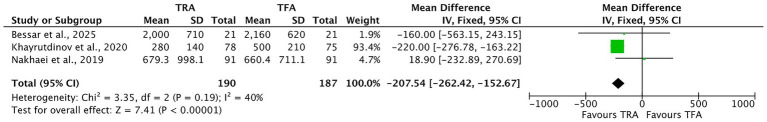
Forest plot comparing radiation exposure between transradial access (TRA) and transfemoral access (TFA). Mean differences (MDs) in dose (mGy) with 95% confidence intervals (CIs) are shown. The TRA group was associated with significantly lower radiation exposure compared to the TFA group. Moderate heterogeneity was observed. A negative mean difference favors the TRA.

Four of the included studies reported the peak radiation dose in milligray (mGy) (Table 2) (13, 14, 16, 18). However, Ghosh et al. (14) did not provide standard deviation values, which prevented the inclusion of their data in the meta-analysis. The pooled analysis showed a statistically significant reduction in radiation exposure associated with the TRA approach during UAE, with an MD of −207.54 mGy (95% CI [−262.42, −152.67]; *p* < 0.00001). Moreover, low heterogeneity was observed (*I*^2^ = 40%), which indicates consistency between the studies (Figure 2). In contrast, Ghosh et al. (14) observed no statistically significant difference between the TRA and TFA groups, reporting mean radiation doses of 2,498 mGy and 2,001 mGy, respectively (*p* > 0.05).

**Table 2:** Procedure-related factors.

Article	Radiation exposure (mGy), mean ± SD		Procedure time (min), mean ± SD		Fluoroscopy duration (min), mean ± SD		Number of patients discharged on the day of the procedure, *N*		Number of failed TRAs that were converted into TFAs
	TRA	TFA	TRA	TFA	TRA	TFA	TRA	TFA	
Bessar et al. 2025 (13)	2000 ± 710	2,160 ± 620	53.95 ± 16.67	65.57 ± 21.58	23.67 ± 8.64	28.05 ± 9.06	NR	NR	0
Ghosh et al. 2022 (14)	2,498	2,001	NR	NR	26	23	NR	NR	0
Gjoreski et al. 2019 (15)	NR	NR	60.3 ± 23.195	72.4 ± 51.5	21.1 ± 10.625	25.3 ± 12.74	11	0	0
Khayrutdinov et al. 2020 (16)	280 ± 140	500 ± 210	32.27 ± 7.99	39.24 ± 9.72	NR	NR	NR	NR	0
Mortensen et al. 2018 (17)	NR	NR	NR	NR	20.12 ± 7.67	20.36 ± 9.48	0	0	0
Nakhaei et al. 2019 (18)	679.3 ± 998.1	660.4 ± 711.1	163.3 ± 38.4	177.1 ± 93.9	39.8 ± 13.6	41.1 ± 16.0	30	4	1

TRA: transradial artery, TFA: transfemoral artery, NR: not reported.

### Procedure time

**Figure 3:**
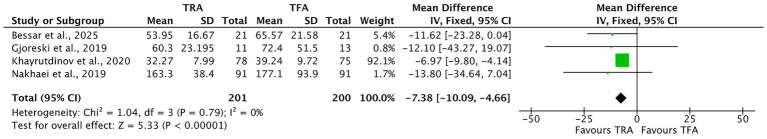
Forest plot comparing procedure times between transradial access (TRA) and transfemoral access (TFA). Mean differences (MDs) with 95% confidence intervals (CIs) are shown. The TRA group demonstrated significantly shorter procedure times than the TFA group. No significant heterogeneity was observed (*I*^2^ = 0%). A negative mean difference favors the TRA.

Four of the included studies reported the mean duration of the UAE procedure for both the TRA and TFA approaches (13, 15, 16, 18). The meta-analysis revealed a statistically significant reduction in procedure time associated with the TRA route, with an MD of −7.38 min (95% CI [−10.09, −4.66], *p* < 0.00001). No heterogeneity was observed (*I*^2^ = 0%), which indicates consistency between the studies (Figure 3).

### Fluoroscopy time

**Figure 4:**
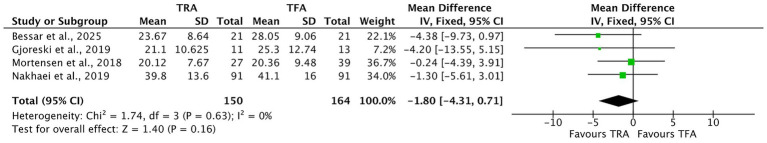
Forest plot comparing fluoroscopy time between transradial access (TRA) and transfemoral access (TFA) in uterine artery embolization. Mean differences (MDs) with 95% confidence intervals (CIs) are shown. No significant difference was observed between the TRA and TFA groups. No significant heterogeneity was detected. A mean difference less than 0 favors the TRA group.

Five of the included studies reported the mean fluoroscopy time (in minutes) for the UAE procedure using both the TRA and TFA approaches (Table 2) (13, 15, 17, 18). However, due to the absence of standard deviation values, the study by Ghosh et al. (14) could not be incorporated into the meta-analysis. Although the majority of studies indicated a shorter fluoroscopy duration with the TRA approach, the pooled analysis did not reveal a statistically significant difference, with an MD of −1.80 (95% CI [−4.31, 0.71], *p* = 0.16). No heterogeneity was observed (*I*^2^ = 0%), indicating consistency across the studies (Figure 4). Similarly, Ghosh et al. (14) found no significant difference in fluoroscopy time between the TRA and TFA groups (26 min vs. 23 min, *p* > 0.05).

### Same-day discharge

**Figure 5:**
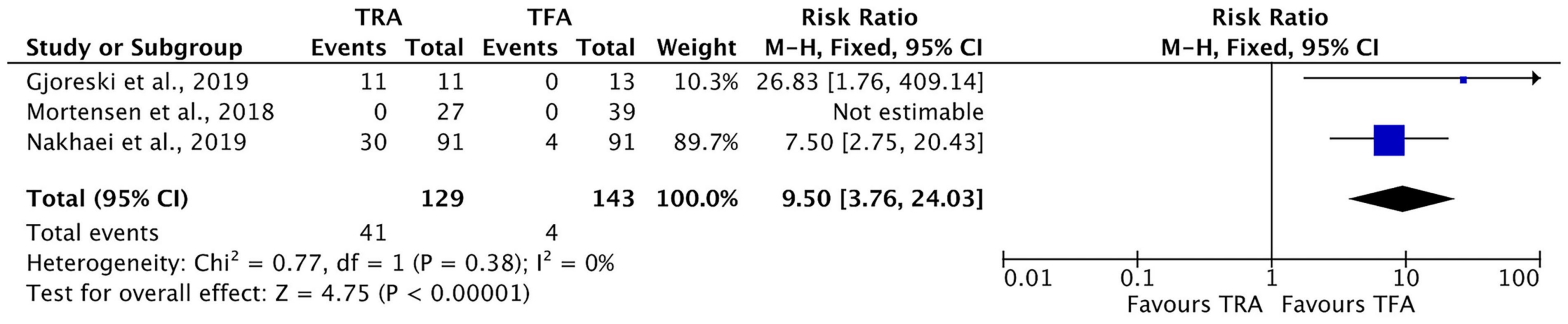
Forest plot comparing same-day discharge rates between transradial access (TRA) and transfemoral access (TFA) in uterine artery embolization. Risk ratios (RRs) with 95% confidence intervals (CIs) are shown. A significantly higher likelihood of same-day discharge was observed in the TRA group than in the TFA group. One study could not be estimated due to zero events in both groups. No significant heterogeneity was observed (*I*^2^ = 0%). A risk ratio greater than 1 favors the TRA.

Three of the included studies reported the number of patients who were discharged on the same day of the UAE procedure (15, 17, 18). Among the 129 patients in the TRA group, 41 (31.78%) were discharged on the same day, compared to 4 of the 143 patients (2.79%) in the TFA group. The meta-analysis revealed a statistically significant association between the TRA approach and an increased likelihood of same-day discharge, with a risk ratio (RR) of 9.50 (95% CI [3.76, 24.03], *p* < 0.00001). No heterogeneity was observed across the studies (*I*^2^ = 0%), indicating high consistency (Figure 5).

### Failed TRA access

All of the included studies reported the successful completion of the UAE procedure through both the TRA and TFA routes (Table 2), with the exception of a single case documented by Nakhaei et al. (18), in which the TRA approach was converted to TFA due to vasospasm. Accordingly, the failure rate for TRA access was 0.3%.

### Postoperative complications

**Figure 6:**
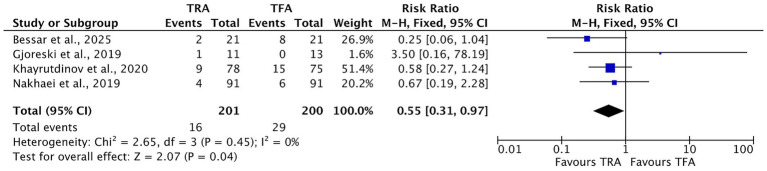
Forest plot comparing access-site complications, such as hematoma, local color changes, arm or groin pain, pseudoaneurysm, and radial artery occlusion, between transradial access (TRA) and transfemoral access (TFA) in uterine artery embolization for fibroids. Risk isk ratios (RRs) with 95% confidence intervals (CIs) are shown. A risk ratio less than 1 favors the TRA. No significant heterogeneity was observed across the subgroups (*I*^2^ = 0%).

Four studies reported postoperative complications following UAE performed through both the TRA and TFA access routes (Table 3) (13, 15, 16, 18). The overall rate of access-site complications, which included hematoma, local discoloration, arm or groin pain, pseudoaneurysm, and radial artery occlusion, was 7.96% in the TRA group compared to 14.5% in the TFA group (Figure 6). The meta-analysis revealed a statistically significant reduction in access-site complications associated with the TRA compared to the TFA (RR = 0.55, 95% CI [0.31, 0.97], *p* = 0.04), with no observed heterogeneity across the studies (*I*^2^ = 0%), indicating consistent findings.

**Table 3:** Postoperative complications.

Article	Total access-site complications		Access-site hematoma		Local color changes		Arm/groin pain		Pseudoaneurysm		Radial artery occlusion	Deep vein thrombosis		Abdominopelvic pain	
	TRA	TFA	TRA	TFA	TRA	TFA	TRA	TFA	TRA	TFA	TRA	TRA	TFA	TRA	TFA
Bessar et al. 2025 (13)	2	8	0	2	2	6	NR	NR	NR	NR	NR	NR	NR	NR	NR
Gjoreski et al. 2019 (15)	1	0	NR	NR	NR	NR	1	0	NR	NR	NR	NR	NR	NR	NR
Khayrutdinov et al. 2020 (16)	9	15	6	13	NR	NR	NR	NR	0	2	3	NR	NR	NR	NR
Nakhaei et al. 2019 (18)	4	6	0	5	NR	NR	0	1	NR	NR	4	1	1	6	7

TRA: transradial artery, TFA: transfemoral artery, NR: not reported.

**Figure 7:**
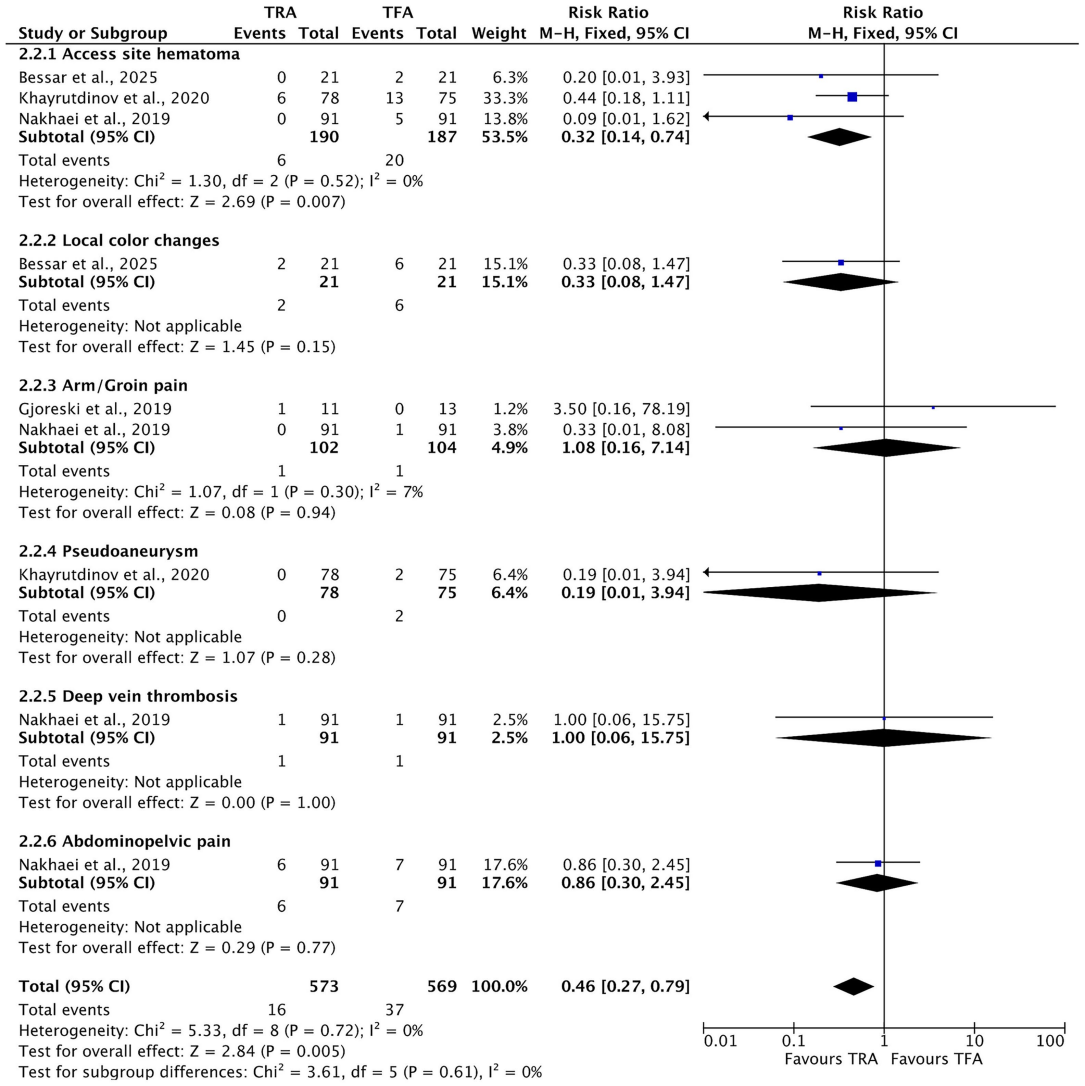
Forest plot of subgroup analysis comparing specific post-procedural complications between transradial access (TRA) and transfemoral access (TFA) in uterine artery embolization. Outcomes utcomes analyzed include both access-site complications, such as hematoma, local color changes, arm/groin pain, and pseudoaneurysm, and non-access-site complications, such as deep vein thrombosis and abdominopelvic pain. Risk ratios (RRs) with 95% confidence intervals (CIs) are shown. A risk ratio less than 1 favors the TRA. No significant heterogeneity was observed across the subgroups (*I*^2^ = 0%).

To further evaluate individual complications, a subgroup analysis was conducted and is presented in Figure 7. This analysis included both access-site complications and other post-procedural events. The TRA was significantly associated with a lower risk of access-site hematoma (RR = 0.32, 95% CI [0.14, 0.74], *p* = 0.007). A trend toward fewer local color changes was observed in the TRA group, although the difference did not reach statistical significance (RR = 0.33, 95% CI [0.08, 1.47], *p* = 0.15). No significant differences were found between the two approaches for arm or groin pain (RR = 1.08, 95% CI [0.16, 7.14], *p* = 0.94), pseudoaneurysm formation (RR = 0.19, 95% CI [0.01, 3.94], *p* = 0.28), deep vein thrombosis (RR = 1.00, 95% CI [0.06, 15.75], *p* = 1.00), or abdominopelvic pain (RR = 0.86, 95% CI [0.30, 2.45], *p* = 0.77). After excluding radial artery occlusion, which was not applicable to the TFA group, the overall complication rate remained significantly lower with the TRA (RR = 0.46, 95% CI [0.27, 0.79], *p* = 0.005), with no observed heterogeneity (*I*^2^ = 0%).

### Risk of bias assessment

The risk of bias of the included randomized studies was assessed independently by two reviewers using the RoB 2 tool. The overall risk of bias of the two RCTs was judged as “high risk” and “some concerns” (Table 4). Non-randomized studies were assessed using the MINORS tool. The total scores ranged from 15 to 18, with a mean score of 17, indicating moderate-to-high quality (Table 5). The lowest-scoring items were the prospective calculation of study size (score of 0 across all the studies) and the unbiased assessment of endpoints (score of 0 to 1). Clearly stated aims, appropriate endpoints, and adequate control groups consistently achieved maximum scores of 2.

**Table 4:** Review of the authors’ judgments about each risk of bias item in each included study, according to the revised Cochrane risk of bias tool (RoB 2).

Article	Bias arising from the randomization process	Bias due to deviations from intended interventions	Bias due to missing outcome data	Bias in the measurement of the outcome	Bias in the selection of the reported result	Overall RoB
Bessar et al. 2025 (13)	Some concerns	Low risk	Low risk	Low risk	Some concerns	Some concerns
Khayrutdinov et al. 2020 (16)	Some concerns	Low risk	Low risk	High risk	Some concerns	High risk

**Table 5:** Review of the authors’ judgments about each risk of bias item in each included study, according to the methodological index for non-randomized studies (MINORS) tool.

Item	Ghosh et al. 2022 (14)	Gjoreski et al. 2019 (15)	Mortensen et al. 2018 (17)	Nakhaei et al. 2019 (18)
A clearly stated aim	2	2	2	2
Inclusion of consecutive patients	1	1	2	2
Prospective collection of data	1	1	2	1
Endpoints appropriate to the aim of the study	2	2	2	2
Unbiased assessment of the study endpoint	1	0	1	1
Follow-up period appropriate to the aim of the study	1	1	1	2
Loss to follow-up less than 5%	2	2	2	1
Prospective calculation of the study size	0	0	0	0
An adequate control group	2	2	**2**	2
Contemporary groups	2	1	1	1
Baseline equivalence of groups	2	2	2	2
Adequate statistical analyses	2	1	2	2
Total score	18	15	17	18

## Discussion

This systematic review and meta-analysis, combining two RCTs, one prospective cohort study, and three retrospective studies, revealed that the TRA confers several clinically meaningful advantages over the traditional TFA for UAE in fibroid management (1–6). However, these findings should be interpreted in the context of study heterogeneity and evolving institutional practice patterns, underscoring the importance of cautious generalization.

The TRA reduced peak skin dose by a mean of 207.5 mGy (*p* < 0.00001), representing a substantial reduction in radiation burden. These findings align with the results of other interventional radiology (IR) procedures, such as prostatic artery embolization, where Richardson et al. reported significantly lower radiation exposure associated with the TRA compared to the TFA, with a mean dose area product (DAP) of 20,705.0 ± 15,962.3 μGy m^2^ versus 31,833.2 ± 22,124.3 μGy m^2^ (*p* < 0.01) (19). However, these results contrast with reports from other procedural domains, particularly coronary angiography and percutaneous coronary intervention, in which the TRA has been associated with higher or neutral radiation doses compared with the TFA (20). This apparent discrepancy, sometimes referred to as the “radiation paradox,” likely reflects differences in procedural complexity, vascular anatomy, and operator experience rather than an inherent radiation-sparing property of radial access itself (20, 21). UAE is typically characterized by predictable pelvic arterial targets and standardized catheter pathways (5), which may reduce the need for extensive catheter exchanges compared with more anatomically variable coronary or neurovascular interventions, particularly once operator proficiency is achieved. Across the included studies, procedures were generally performed by experienced interventional radiologists; however, the degree of specific experience with the TRA varied, with several studies encompassing early adoption phases. As such, the observed reductions in radiation exposure may be influenced by operator skill and institutional practice patterns and may not be immediately generalizable to centers in the initial learning curve.

Furthermore, the TRA was associated with significantly shorter surgical procedure times. This analysis showed an average reduction of 7.38 min (*p* < 0.00001), with no variation among the studies (*I*^2^ = 0%). Although baseline times varied between studies, this reduction may improve workflow efficiency and reduce anesthesia duration. Eldeeb et al. (22) conducted a systematic review and meta-analysis on transarterial radioembolization (TARE) for liver tumors. Although not limited to UAE, their findings similarly showed that the TRA was associated with a significant reduction in procedural duration (MD − 6.3 min; *p* = 0.005) without increased radiation exposure, indicating efficiency benefits consistent with our results.

The likelihood of same-day discharge was significantly higher in the TRA group, with 31.8% of patients discharged the same day compared to only 2.8% in the TFA group (*p* < 0.00001), highlighting a substantial improvement in recovery and hospital resource utilization. This 29-percentage-point absolute gain translates into fewer inpatient bed-hours and aligns with interventional radiology series showing TRA as a key enabler of outpatient embolization pathways. A study by Sher et al. reported that 97.9% of patients undergoing UAE through the TRA were discharged the same day, with low rates of unplanned clinic visits (3.2%), emergency department visits (5.1%), and readmissions (0.5%) within 30 days (23). While these results support the feasibility and advantages of same-day discharge following the TRA, it is important to recognize that institutional protocols and patient selection criteria significantly influence discharge practices. Therefore, the adoption of TRA should be tailored to the specific circumstances of each patient and institution.

The overall access-site complication rate decreased from 14.5% with the TFA to 8.0% with the TRA (*p* = 0.04), driven chiefly by a reduction in groin hematomas (RR = 0.32). There was no significant increase in pain, pseudoaneurysm, or deep vein thrombosis. Such findings have encouraged using the TRA more commonly in settings that value patient mobility, comfort, and quicker discharge. Our results align with the findings of a study by Posham et al. examining non-coronary interventions, which concluded that the TRA significantly reduces access-site complications without compromising procedural success (24).

While the TRA reduces femoral access-site complications, it introduces a unique risk of radial artery occlusion (RAO). In the included studies, RAO was infrequently reported and, when observed, was transient and managed conservatively without long-term sequelae (18). Several studies explicitly acknowledged this risk and described preventive strategies, including radial artery assessment, use of smaller-caliber sheaths, intraprocedural anticoagulation, and non-occlusive patent hemostasis (16, 17). Although uncommon, RAO represents a transradial-specific trade-off that should be discussed during procedural planning and patient counseling.

Operator experience remains a critical determinant of TRA outcomes. Evidence from non-coronary endovascular literature suggests that the adoption of transradial techniques is associated with an initial learning phase during which procedure time and radiation exposure may increase (17, 24). In the included studies, procedures were generally performed by experienced interventional radiologists, although the degree of specific transradial expertise varied, and several studies included the early adoption phases. Consequently, the favorable procedural metrics observed may reflect operator skill and institutional experience and may not be immediately reproducible during early implementation, highlighting the importance of structured training and appropriate case selection.

Nonetheless, the TFA continues to play an indispensable role in clinical practice. It remains the preferred route in complex anatomical settings, including in patients with radial artery spasm, severe tortuosity, subclavian stenosis, or aberrant aortic arch anatomy (25). Additionally, the TFA is typically necessary for procedures requiring large-bore access or stent delivery systems, where radial artery caliber may be inadequate (26).

Despite the strengths of our systematic review, several limitations should be acknowledged. The number of studies was not large (*N* = 6), of which only two were RCTs. The remaining studies were observational, which could result in selection bias and limit the strength of causal inferences. Additionally, individual studies used small samples, which reduced the ability to detect infrequent outcomes, such as rare complications. Moreover, differences in reporting methods made the analysis more difficult. One of the studies (Ghosh et al. (14)) did not include important statistics, such as standard deviation (SD) for radiation exposure or fluoroscopy time, which prevented them from being included in the meta-analyses, thereby reducing the overall statistical strength. Moreover, the degree of expertise among the operators and the procedures used in each study may have influenced the outcomes, particularly for the TRA, which has a well-known learning curve. The demographic profile of participants was rather homogeneous, consisting primarily of middle-aged women, limiting generalizability to other populations with varied anatomical or clinical features.

Importantly, long-term, patient-centered outcomes, including symptom recurrence, the need for reintervention, fertility outcomes, and standardized quality-of-life measures, were inconsistently reported or not systematically assessed across the included studies. Consequently, this meta-analysis primarily reflects short-term procedural and access-site outcomes. Future research should therefore focus on large, multicenter randomized controlled trials with standardized outcome reporting and planned long-term follow-up. Additional studies evaluating patient-centered outcomes such as recovery experience, patient satisfaction, and cost-effectiveness would further inform clinical decision-making and support the broader implementation of TRA where appropriate.

## Conclusion

TRA is a safe and effective alternative to the TFA for UAE. It offers reduced radiation exposure, shorter procedure times, fewer access-site complications, and higher same-day discharge rates. While the TFA remains useful in select cases, the TRA may improve patient outcomes and procedural efficiency.

## Data Availability

The original contributions presented in the study are included in the article/supplementary material, further inquiries can be directed to the corresponding author.
