# Correction: Transradial versus transfemoral access in uterine artery embolization for fibroids: a systematic review and meta-analysis

**DOI:** 10.3389/fmed.2026.1830166

**Published:** 2026-03-25

**Authors:** Frontiers Production Office

**Affiliations:** Frontiers Media SA, Lausanne, Switzerland

**Keywords:** interventional radiology, minimally invasive therapy, transfemoral access, transradial access, uterine artery embolization, uterine fibroids

Author affiliations and superscripts were not updated and were erroneously listed as:

Zainah Abdulbari Alhebshi 1^*^, Marwah Nasir Ahmad 1, Mariam Amro Alsayed 1, Layan Hassan Aljarari 1, Maya Rawah 1, Mahnoor Hayat 1, Jina Khalid Mohammed Fadl 2,3, Ibrahim Daoud 2,4, Omnia Orabi Mohammed Orabi 2,5 and Salma Mohammed Hassan Eltayeb 6

1. General Medicine Practice Program, Batterjee Medical College, Jeddah, Saudi Arabia

2. Department of Obstetrics and Gynecology, General Medicine Practice Program, Batterjee Medical College, Jeddah, Saudi Arabia

3. Faculty of Medicine, University of Khartoum, Khartoum, Sudan

4. Faculty of Medicine, Al Neelain University, Khartoum, Sudan

5. Faculty of Medicine, Suez Canal University, Ismailia, Egypt

6. Department of Obstetrics and Gynecology, Tawam Hospital, AlAin, United Arab Emirates

The correct version is:

Zainah Abdulbari Alhebshi^1*^, Marwah Nasir Ahmad^1^, Mariam Amro Alsayed^1^, Layan Hassan Aljarari^1^, Maya Rawah^1^, Mahnoor Hayat^1^, Jina Khalid Mohammed Fadl^2, 3^, Ibrahim Daoud^4, 5^, Omnia Orabi Mohammed Orabi^2, 6^ and Salma Mohammed Hassan Eltayeb^7^

^1^General Medicine Practice Program, Batterjee Medical College, Jeddah, Saudi Arabia

^2^Department of Obstetrics and Gynecology, General Medicine Practice Program, Batterjee Medical College, Jeddah, Saudi Arabia

^3^Faculty of Medicine, University of Khartoum, Khartoum, Sudan

^4^Department of Obstetrics and Gynecology, General Medicine Practice Program, Batterjee Medical College, Aseer, Saudi Arabia

^5^Faculty of Medicine, Al Neelain University, Khartoum, Sudan

^6^Faculty of Medicine, Suez Canal University, Ismailia, Egypt

^7^Department of Obstetrics and Gynecology, Tawam Hospital, AlAin, United Arab Emirates

The original version of this article has been updated.

